# Reversible elevation of creatine kinase and creatinine caused by sintilimab-induced hypothyroidism: A case report

**DOI:** 10.1097/MD.0000000000040080

**Published:** 2024-10-18

**Authors:** Shu-Rong Liu, Zhen-Guang Zhao, Rui-Ren Zhai, Li-Juan Wang, Chen Yang, Quan-Bin Ma, Li Wang

**Affiliations:** a Department of Oncology, Sunshine Union Hospital, Weifang, Shandong Province, China; b Department of Gastroenterology, Beijing Puxiang Hospital Of TCM, Beijing, China; c Department of Hematology and Oncology, Laoshan Medical District of No. 971 Hospital of Chinese Navy, Qingdao, Shandong Province, China.

**Keywords:** case report, creatine kinase, creatinine, hypothyroidism, immune-related adverse events, programmed cell death-1 inhibitors, sintilimab

## Abstract

**Rationale:**

Programmed cell death (PD) -1 inhibitors has significantly improved the prognosis of cancer patients by enhancing antitumor immune responses. However, PD-1 inhibitors are associated with immune-related adverse events, some of which are rare and potentially life-threatening. Thus far, elevated creatine kinase (CK) and creatinine caused by a novel PD-1 inhibitor (sintilimab)-induced hypothyroidism has not yet been reported.

**Patient concerns:**

A 63-year-old male patient with esophageal cancer who developed hypothyroidism accompanied by unexplained increases in CK and creatinine after sintilimab treatment.

**Diagnosis:**

Since the increases in CK and creatinine paralleled the decrease in thyroxine, after excluding other potential conditions, we speculated that the muscular and renal dysfunction might be caused by sintilimab-induced hypothyroidism.

**Interventions and outcomes:**

As the patient’s thyroid function improved with levothyroxine replacement therapy, the levels of CK and creatinine concomitantly returned to normal.

**Conclusion and lessons:**

The elevated CK and creatinine levels in this patient were caused by sintilimab-induced hypothyroidism. Our case highlights the importance of keeping PD-1 induced hypothyroidism in mind when patients present with unexplained increased levels of CK and creatinine. Hypothyroidism-related muscular and renal dysfunctions, which can be restored with thyroid hormone replacement, need to be identified early and treated promptly so that unnecessary examinations and treatments can be avoided in these patients.

## 1. Introduction

Programmed cell death (PD) -1 inhibitors can block the binding of the PD-1 receptor and PD ligand 1, thus reactivating T lymphocyte function and enhancing antitumor immune response.^[1]^ PD-1 inhibitors have profoundly improved the prognosis of patients with malignant tumors.^[2,3]^ PD-1 inhibitors are generally well tolerated, but are associated with immune-related adverse events (irAEs).^[4]^ Thyroid dysfunction is 1 of the most frequent irAEs after PD-1 treatment.^[4,5]^ Although the underlying mechanisms of PD-1 related thyroid dysfunction are still unclear, the activation of B cells via T-cell dependent pathways and the subsequent release of antibodies may be involved in this pathophysiological process.^[1]^

Sintilimab, a novel PD-1 inhibitor, has been widely used in various malignant tumors, and is associated with impressive outcomes.^[6–10]^ Here, we present the first case of reversible elevated creatine kinase (CK) and creatinine caused by sintilimab-induced hypothyroidism. As thyroid function improved with levothyroxine replacement therapy, the CK and creatinine levels returned to normal. Our case highlights the importance of early identification of muscular and renal dysfunctions caused by PD-1-induced hypothyroidism, which can be rectified with thyroid hormone replacement, allowing these patients to avoid unnecessary examinations and treatments.

## 2. Case description

Ethics committee of Sunshine Union Hospital approved this study. Written informed consent was obtained from the patient. A 63-year-old male patient was diagnosed with esophageal squamous cell carcinoma. After 2 cycles of neoadjuvant chemotherapy (paclitaxel plus cisplatin) in combination with sintilimab (200 mg/Q3w), radical resection of esophageal cancer was performed, and postoperative pathology confirmed the initial diagnosis. One month after the surgery, postoperative adjuvant chemotherapy (paclitaxel plus cisplatin) combined with sintilimab was administered.

**Figure 1.**
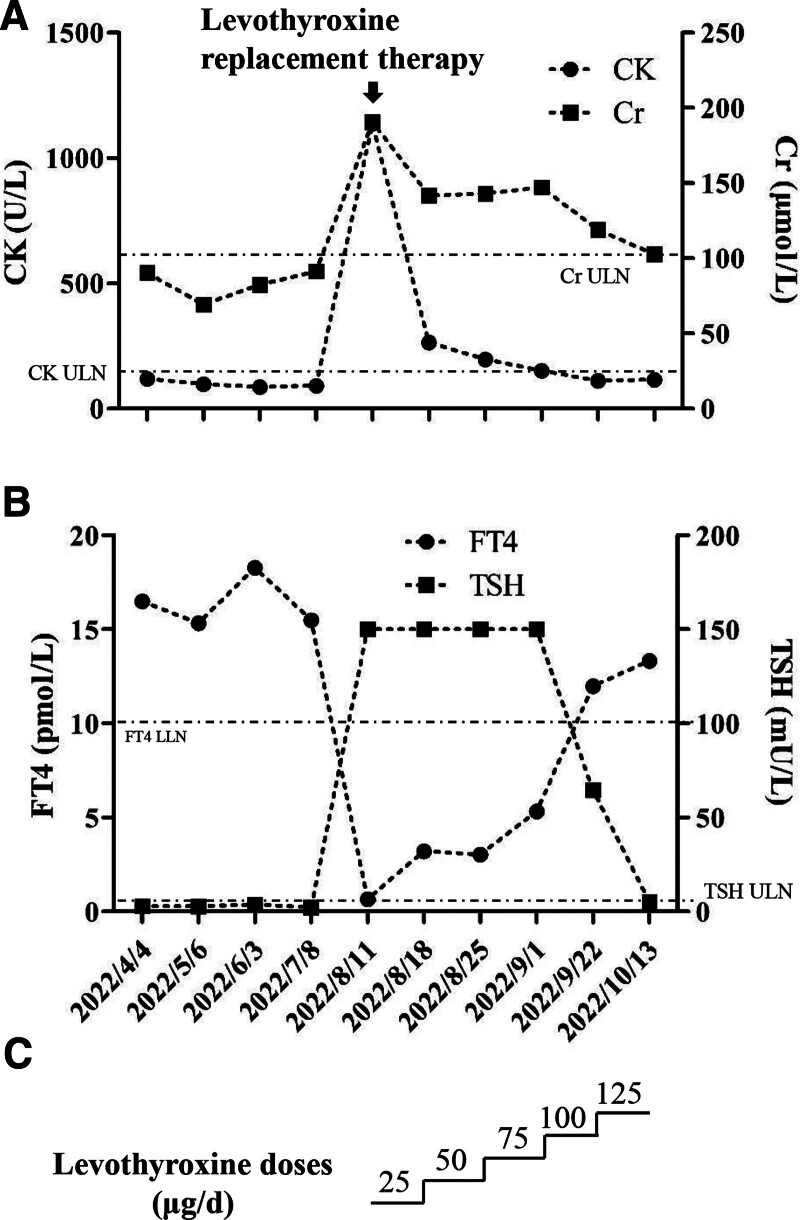
Levels of CK, Cr, FT4 and TSH before and after levothyroxine replacement therapy. (A) levels of CK and Cr; (B) levels of FT4 and TSH; (C) levothyroxine replacement regimens. CK = creatine kinase, Cr = creatinine, FT4 = free thyroxine, LLN = low limit of normal, TSH = thyroid stimulating hormone, ULN = upper limit of normal.

Approximately 4 months after the initiation of sintilimab, after the first cycle of postoperative chemotherapy, the patient developed mild muscular soreness and fatigue. Laboratory examination revealed that CK (from 119.3 to 1136.7 U/L, normal range: 2–200 U/L) and creatinine (from 90.4 to 190.5 μmol/L, normal range: 57–97 μmol/L) levels markedly increased (Fig. 1A). The estimated glomerular filtration rate decreased from 78.1 to 33.1 ml/min/1.73m^2^.

The levels of myoglobin, cardiac troponin T, and CK-MB were normal. Antinuclear antibodies and anti-extractable nuclear antigen antibodies (including anti-Sm, anti-SS-A/Ro, SS-B/La, anti-Scl-70, anti-Jo-1, anti-U1-RNP, and anti-r-RNP) were all negative. Urinalysis revealed no proteinuria or hematuria. Cortisol and adrenocorticotropic hormone levels were normal. Electrocardiogram and echocardiography examinations showed no abnormalities. Muscular biopsy revealed no obvious inflammatory presentation except for mild myogenic changes.

Thyroid function tests indicated hypothyroidism (Table 1, Fig. 1B), and his levels of antithyroid peroxidase antibodies and antithyroglobulin antibodies were normal. The patient had no prior history of any thyroid disease, and his levels of T3, T4, FT3, FT4, and TSH were normal prior to the initiation of sintilimab and up to 4 months before the presentation of symptoms (Table 1). Thus, the patient was diagnosed with sintilimab-induced hypothyroidism. Since the increases in CK and creatinine paralleled the decrease in thyroxine, after excluding other potential conditions, we hypothesized that there might be an association between elevated CK and creatinine and hypothyroidism.

**Table 1:** Thyroid function tests before and after levothyroxine replacement in this patient.

Date	T3 (nmol/L)	T4 (nmol/L)	FT3 (pmol/L)	FT4 (pmol/L)	TSH (mU/L)
April 4, 2022*	2.00	128.5	5.03	16.49	2.90
May 6, 2022	2.13	142.3	4.98	15.32	2.75
June 3, 2022	2.35	158.1	5.36	18.27	3.81
July 8, 2022	2.02	136.7	4.71	15.49	2.10
August 11, 2022#	<0.15	9.3	<0.3	0.66	>150.0
August 18, 2022	0.25	27.2	1.04	3.20	>150.0
August 25, 2022	0.24	29.1	1.23	3.02	>150.0
September 1, 2022	0.38	43.1	2.05	5.32	>150.0
September 22, 2022	1.35	72.9	3.39	11.97	64.59
October 13, 2022	1.79	108.8	4.23	13.33	5.28

FT3 = free triiodothyronine, FT4 = free thyroxine, T3 = triiodothyronine, T4 = thyroxine, TSH = thyroid stimulating hormone.

* Represents the baseline thyroid functions.

# Represents the initiation of levothyroxine replacement therapy.

Then, levothyroxine at a dose of 25 μg daily was orally administered. The doses were gradually increased to 125 μg daily (Fig. 1C). As the levels of T3, T4, FT3, FT4, and TSH gradually improved (Table 1, Fig. 1B), the CK level dramatically decreased (from 1136.7 to 264 U/L in 1 week), and the creatinine level also gradually returned to normal (Fig. 1A). The symptoms of muscular soreness and fatigue gradually disappeared. Then, a second cycle of postoperative chemotherapy in combination with sintilimab was given. The thyroid function test results and CK and creatinine levels remained normal.

## 3. Discussion

To the best of our knowledge, this is the first case of elevated CK and creatinine caused by sintilimab-induced hypothyroidism. In this case, the patient developed hypothyroidism 4 months after the initiation of sintilimab treatment, accompanied by unexplained increases in CK and creatinine. With thyroid hormone replacement only, the increased CK and creatinine returned to normal as the patient’s thyroid function improved. Hypothyroidism is 1 of the most common irAEs after sintilimab treatment. Based on published data (Table 1, Supplemental Digital Content, http://links.lww.com/MD/N731, which illustrates characteristics of clinical studies reporting the frequencies of hypothyroidism after sintilimab treatment), our pooled analysis revealed that approximately 15% of patients experienced hypothyroidism after sintilimab treatment (Figure 1, Supplemental Digital Content, http://links.lww.com/MD/N731, which illustrates forest plot of hypothyroidism after sintilimab treatment). Therefore, closely monitoring thyroid function and related muscular and renal dysfunctions after sintilimab treatment is highly recommended.

Differential diagnoses of hypothyroidism related to other diseases caused elevated CK and/or creatinine are required,^[11]^ specifically myocarditis, which is associated with a high mortality rate.^[11–13]^ In this patient, the levels of myoglobin, cardiac troponin T, and CK-MB were normal, excluding the possibility of myocarditis. However, differentiating hypothyroidism-related renal dysfunction from PD-1-induced glomerular or tubulointerstitial nephritis is clinically difficult. Renal biopsies were recommended, but this patient refused this invasive procedure. Since the increase in CK and creatinine paralleled the decrease in thyroxine, we speculated that there might be a correlation between elevated CK and creatinine and hypothyroidism. Fortunately, with levothyroxine replacement therapy without steroid treatment, as thyroid function improved, the levels of CK and creatinine concomitantly returned to normal. Therefore, we confirmed the hypothesis that the elevated CK and creatinine levels in this patient were caused by sintilimab-induced hypothyroidism.

Elevated CK and creatinine caused by hypothyroidism can be restored by levothyroxine replacement therapy. However, in some cases, even if high doses of levothyroxine are administered, the myopathic symptoms might persist, possibly due to decreased intramuscular deiodination of T4 to T3. Coadministration of T4 and T3 might be beneficial in these patients.^[14]^ In addition, in some cases, steroids were used, which was unavoidable due to difficulties in differential diagnoses. Previous studies indicated that a small portion of PD-1-induced hypothyroidism could be rescued by steroid treatment, and patients exposed to steroids could recover with relatively lower doses of levothyroxine.^[15]^ However, we still recommend precise treatment for hypothyroidism because of the side effects related to steroids. Notably, it is essential to rule out adrenal insufficiency before levothyroxine replacement because the inhibitory effect of levothyroxine replacement on the hypothalamic–pituitary–adrenal axis may trigger an adrenal crisis.^[16]^

Several issues should be considered. Notably, PD-1-induced hyperthyroidism sometimes occurs prior to hypothyroidism. Hyperthyroidism is often caused by the destruction of thyroid cells, lasts for a short time, and ultimately progresses to hypothyroidism.^[1]^ Second, elevated CK and/or creatinine caused by PD-1-induced hypothyroidism has also been reported for other types of PD-1 inhibitors,^[16,17]^ indicating that clinicians should pay more attention to the adverse effects related to PD-1 inhibitors. However, due to the small number of related cases, the underlying mechanisms behind PD-1-induced hypothyroid-induced elevated CK and creatinine need to be clarified in further studies. Finally, PD-1 inhibitors are usually not discontinued as the symptoms are managed with thyroid hormone replacement.^[18]^ Moreover, immune-related hypothyroidism might indicate stronger antitumor immune responses, suggesting that these episodes might be beneficial to patients.^[1,19–21]^ With prompt recognition and appropriate treatment, patients with PD-1-induced hypothyroidism might have better clinical outcomes.

In conclusion, with the increasing uses of PD-1 inhibitors in a variety of malignant tumors, similar cases of PD-1-induced hypothyroidism could occur more commonly. Our case highlights the importance of taking PD-1-induced hypothyroidism into consideration when patients present with unexplained elevated levels of CK and creatinine. Hypothyroidism-related muscular and renal dysfunctions, which can be restored with thyroid hormone replacement, need to be identified early and treated promptly so that unnecessary examinations and treatments can be avoided in these patients.

## Author contributions

**Conceptualization:** Li Wang.

**Data curation:** Shu-Rong Liu, Zhen-guang Zhao, Li-Juan Wang, Chen Yang, Quan-Bin Ma, Li Wang.

**Investigation:** Shu-Rong Liu, Rui-Ren Zhai, Li-Juan Wang, Chen Yang, Quan-Bin Ma, Li Wang.

**Methodology:** Shu-Rong Liu, Zhen-guang Zhao, Rui-Ren Zhai, Li-Juan Wang, Chen Yang, Quan-Bin Ma, Li Wang.

**Resources:** Li Wang.

**Supervision:** Zhen-Guang Zhao, Chen Yang, Li Wang.

**Validation:** Shu-Rong Liu, Li Wang.

**Visualization:** Li Wang.

**Writing – original draft:** Shu-Rong Liu, Zhen-guang Zhao, Chen Yang, Li Wang.

**Writing – review & editing:** Shu-Rong Liu, Zhen-Guang Zhao, Rui-Ren Zhai, Li-Juan Wang, Chen Yang, Quan-bin Ma, Li Wang.

## Supplementary Material


