# Supplementary material for: Reversible elevation of creatine kinase and creatinine caused by sintilimab-induced hypothyroidism: A case report
Source: Medicine (Baltimore). 2024 Oct 18;103(42):e40080. doi: 10.1097/MD.0000000000040080 (PMC11495781; doi:10.1097/MD.0000000000040080)
Supplement: Supplementary file 1 [file medi-103-e40080-s001.docx]

**Reversible** **elevation of** **creatine kinase and creatinine caused by sintilimab-induced hypothyroidism: A case report**

Li Wang et al

**Supplementary Table 1**. Characteristics of clinical studies reporting the frequencies of hypothyroidism after sintilimab treatment

| **Study** | **Published year** | **Diagnosis** | **Sample size** | **Hypothyroidism numbers (rates)** | **Hypothyroidism grades** |
| --- | --- | --- | --- | --- | --- |
| Wang et al^1^ | 2024 | Hepatocellular carcinoma | 97 | 4 (4.1%) | Grade 1-2 |
| Xu et al^2^ | 2023 | Gastric  or gastroesophageal junction cancer | 327 | 58 (17.7%) | Grade 1-2 |
| Lu et al^3^ | 2023 | Non-squamous NSCLC | 156 | 17 (10.9%) | Grade 1-2 |
| Wang et al^4^ | 2023 | Cervical cancer | 27 | 5 (18.5%) | Grade 1-2 |
| Gao et al^5^ | 2023 | Gastric cancer | 34 | 8 (23.5%) | Grade 1-2 |
| Lu et al^6^ | 2023 | Nasopharyngeal carcinoma | 33 | 14 (42.4%) | Grade 1-2 |
| Liu et al^7^ | 2023 | Soft tissue sarcoma | 39 | 22 (56.4%) | Grade 1-2 |
| Lin et al^8^ | 2023 | Squamous NSCLC | 52 | 2 (3.8%) | Grade 1-2 |
| Lu et al^9^ | 2022 | Esophageal carcinoma | 327 | 41 (12.5%) | Grade 1-2 |
| Shi et al^10^ | 2022 | Squamous NSCLC | 144 | 21 (14.6%) | Grade 1-2 |
| Guo et al^11^ | 2022 | Gastric cancer | 30 | 1 (3.3%) | Grade 2 |
| Zhao et al^12^ | 2022 | Hepatocellular carcinoma | 60 | 5 (8.3%) | Grade 1 |
| Xu et al^13^ | 2022 | Cervical cancer | 42 | 14 (33.3%) | Grade 2 |
| Li et al^14^ | 2022 | Hepatocellular carcinoma | 46 | 7 (15.2%) | Grade 1-2 |
| Tian et al^15^ | 2022 | Soft tissue sarcoma | 28 | 4 (14.3%) | Grade 1-2 |
| Jin et al^16^ | 2022 | Biliary tract cancer | 20 | 6 (30%) | Grade 1-2 |
| Duan et al^17^ | 2021 | Esophageal cancer | 23 | 2 (8.7%) | Grade 1-2 |
| Ren et al^18^ | 2021 | Hepatocellular carcinoma | 380 | 53 (13.9%) | Grade 1-2 |
| Zhou et al^19^ | 2021 | Squamous NSCLC | 179 | 18 (10.1%) | Grade 1-2 |

Abbreviation: NSCLC: non‑small‑cell lung cancer

**References**

1. Wang K, Xiang YJ, Yu HM, et al. Adjuvant sintilimab in resected high-risk hepatocellular carcinoma: a randomized, controlled, phase 2 trial. *Nature medicine.* 2024.

2. Xu J, Jiang H, Pan Y, et al. Sintilimab plus chemotherapy for unresectable gastric or gastroesophageal junction cancer: The ORIENT-16 randomized clinical trial. *JAMA : the journal of the American Medical Association.* 2023;330(21):2064-2074.

3. Lu S, Wu L, Jian H, et al. Sintilimab plus chemotherapy for patients with EGFR-mutated non-squamous non-small-cell lung cancer with disease progression after EGFR tyrosine-kinase inhibitor therapy (ORIENT-31): second interim analysis from a double-blind, randomised, placebo-controlled, phase 3 trial. *The Lancet. Respiratory medicine.* 2023;11(7):624-636.

4. Wang Y, Zhao J, Liang H, et al. Efficacy and safety of sintilimab plus albumin-bound-paclitaxel in recurrent or metastatic cervical cancer: a multicenter, open-label, single-arm, phase II trial. *EClinicalMedicine.* 2023;65:102274.

5. Gao L, Tang L, Li X, Peng J, Hu Z, Liu B. Efficacy and safety of sintilimab combined with apatinib as third-line or above therapy for patients with advanced or metastatic gastric cancer. *Anti-cancer drugs.* 2023;35(3):277-283.

6. Lu N, Jiang YF, Xia WX, et al. Efficacy and safety of sintilimab plus bevacizumab in metastatic nasopharyngeal carcinoma after failure of platinum-based chemotherapy: an open-label phase 2 study. *EClinicalMedicine.* 2023;62:102136.

7. Liu Z, Wang X, Wang J, et al. The efficacies and biomarker investigations of antiangiogenic agents and PD-1 inhibitors for metastatic soft tissue sarcoma: A multicenter retrospective study. *Frontiers in oncology.* 2023;13:1124517.

8. Lin X, Deng H, Li S, et al. Sintilimab with chemotherapy as first-line treatment for locally advanced or metastatic squamous non-small-cell lung cancer: a real-world data study. *Journal of cancer research and clinical oncology.* 2023;149(2):757-764.

9. Lu Z, Wang J, Shu Y, et al. Sintilimab versus placebo in combination with chemotherapy as first line treatment for locally advanced or metastatic oesophageal squamous cell carcinoma (ORIENT-15): multicentre, randomised, double blind, phase 3 trial. *BMJ (Clinical research ed.).* 2022;377:e068714.

10. Shi Y, Wu L, Yu X, et al. Sintilimab versus docetaxel as second-line treatment in advanced or metastatic squamous non-small-cell lung cancer: an open-label, randomized controlled phase 3 trial (ORIENT-3). *Cancer communications (London, England).* 2022;42(12):1314-1330.

11. Guo H, Ding P, Sun C, et al. Efficacy and safety of sintilimab plus XELOX as a neoadjuvant regimen in patients with locally advanced gastric cancer: A single-arm, open-label, phase II trial. *Frontiers in oncology.* 2022;12:927781.

12. Zhao L, Chang N, Shi L, et al. Lenvatinib plus sintilimab versus lenvatinib monotherapy as first-line treatment for advanced HBV-related hepatocellular carcinoma: A retrospective, real-world study. *Heliyon.* 2022;8(6):e09538.

13. Xu Q, Wang J, Sun Y, et al. Efficacy and Safety of Sintilimab Plus Anlotinib for PD-L1-Positive Recurrent or Metastatic Cervical Cancer: A Multicenter, Single-Arm, Prospective Phase II Trial. *Journal of clinical oncology : official journal of the American Society of Clinical Oncology.* 2022;40(16):1795-1805.

14. Li D, Xu L, Ji J, et al. Sintilimab combined with apatinib plus capecitabine in the treatment of unresectable hepatocellular carcinoma: A prospective, open-label, single-arm, phase II clinical study. *Frontiers in immunology.* 2022;13:944062.

15. Tian Z, Dong S, Yang Y, et al. Nanoparticle albumin-bound paclitaxel and PD-1 inhibitor (sintilimab) combination therapy for soft tissue sarcoma: a retrospective study. *BMC cancer.* 2022;22(1):56.

16. Jin S, Zhao R, Zhou C, et al. Feasibility and tolerability of sintilimab plus anlotinib as the second-line therapy for patients with advanced biliary tract cancers: An open-label, single-arm, phase II clinical trial. *International journal of cancer. Journal international du cancer.* 2023;152(8):1648-1658.

17. Duan H, Wang T, Luo Z, et al. A multicenter single-arm trial of sintilimab in combination with chemotherapy for neoadjuvant treatment of resectable esophageal cancer (SIN-ICE study). *Annals of translational medicine.* 2021;9(22):1700.

18. Ren Z, Xu J, Bai Y, et al. Sintilimab plus a bevacizumab biosimilar (IBI305) versus sorafenib in unresectable hepatocellular carcinoma (ORIENT-32): a randomised, open-label, phase 2-3 study. *The lancet oncology.* 2021;22(7):977-990.

19. Zhou C, Wu L, Fan Y, et al. Sintilimab plus platinum and gemcitabine as first-line treatment for advanced or metastatic squamous NSCLC: results from a randomized, double-blind, phase 3 trial (ORIENT-12). *Journal of thoracic oncology : official publication of the International Association for the Study of Lung Cancer.* 2021;16(9):1501-1511.

**Reversible elevation of creatine kinase and creatinine caused by sintilimab-induced hypothyroidism: A case report**

Li Wang et al


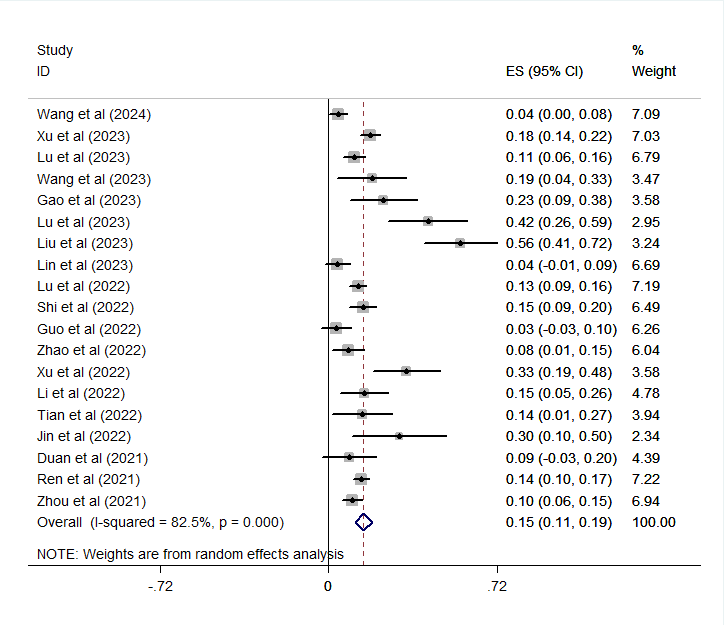


**Supplementary Figure 1**. Forest plot of hypothyroidism after sintilimab treatment.
